# A case report of primary mucinous adenocarcinoma of the bladder and literature review

**DOI:** 10.3389/fonc.2026.1809355

**Published:** 2026-06-22

**Authors:** Yue Li, Xuefeng Peng, Suiyang Jin, Qiang Wang

**Affiliations:** 1 Department of Urology, The Affiliated Hospital of Guizhou Medical University, Guiyang, Guizhou, China; 2 Clinical Medical School, Guizhou Medical University, Guiyang, Guizhou, China

**Keywords:** bladder adenocarcinoma, bladder cancer, mucinous adenocarcinoma, prognosis, treatment methods

## Abstract

Primary mucinous adenocarcinoma of the bladder is a rare and highly aggressive malignancy. Its diagnosis is challenging due to the need to exclude metastatic gastrointestinal adenocarcinoma, and standardized treatment protocols are lacking. Here, we report a case of primary bladder mucinous adenocarcinoma in a 70-year-old female who presented with urinary difficulty without hematuria. The diagnostic workup integrated endoscopic evaluation with immunohistochemical markers (CK7–, CK20+, CDX-2+, β-catenin membrane+, SATB2–, GATA3–) to rule out a colorectal primary. The patient underwent transurethral resection followed by robot-assisted radical cystectomy (pT4aN0M0) but declined adjuvant therapy for personal reasons. At 6-month follow-up, no recurrence or metastasis was detected. This case highlights the diagnostic utility of a targeted immunohistochemical panel, the real-world complexity of treatment decisions in the absence of guidelines, and is compared with all reported cases from the past decade in a structured literature review.

## 1 Introduction

Primary bladder adenocarcinoma is an uncommon malignant tumor. Its histological features are characterized by a pure glandular pattern, accounting for approximately 0.5% to 2% of all bladder cancers (1). Its histopathological subtypes include intestinal, mucinous, signet ring cell, clear cell, undifferentiated, and mixed types (2). Among these, bladder mucinous adenocarcinoma accounts for approximately 20% of primary bladder adenocarcinomas (3). Accurate diagnosis of this entity is particularly challenging, as it must be distinguished from metastatic adenocarcinoma of gastrointestinal origin. This is a critical distinction that carries significant therapeutic and prognostic implications. Recent studies have highlighted the importance of integrating histopathological assessment with a panel of immunohistochemical markers, including CK7, CK20, CDX-2, SATB2, and β-catenin, to clarify tumor origin (4, 5). Moreover, the management of primary bladder mucinous adenocarcinoma is complicated by the lack of standardized treatment protocols, and current clinical decisions are largely informed by limited case reports and retrospective series (6). Given the rarity and diagnostic complexity of this disease, there remains a pressing need for well-documented cases that contribute to the refinement of diagnostic and therapeutic strategies (7).

Here, we report a case of primary mucinous adenocarcinoma of the bladder with three distinctive features that differentiate it from previously published cases. First, the immunohistochemical findings of our patient were as follows: CK7 was negative, whereas CK20, CDX-2, and β-catenin (membranous expression) were positive, and both SATB2 and GATA3 were negative. This immunophenotype, together with the absence of abnormal findings on endoscopic examination, serves as a detailed illustration of the diagnostic reasoning process required to rule out a gastrointestinal primary. Second, the patient received no neoadjuvant therapy before radical cystectomy and, owing to personal considerations, declined any form of postoperative adjuvant treatment. This clinical scenario highlights the practical complexities of therapeutic decision-making when standardized guidelines for such a rare tumor are lacking. Third, we conducted a structured comparative analysis of our case against all documented instances of primary mucinous adenocarcinoma of the bladder reported over the preceding decade. The comparison encompassed clinical presentation, tumor stage, treatment modality, and clinical outcomes, thereby situating our observations within the context of the existing literature. Through this report, we seek to deepen clinicians’ understanding of the diagnostic evaluation and therapeutic considerations for this rare and aggressive bladder malignancy.

## 2 Case report

**Figure 1:**
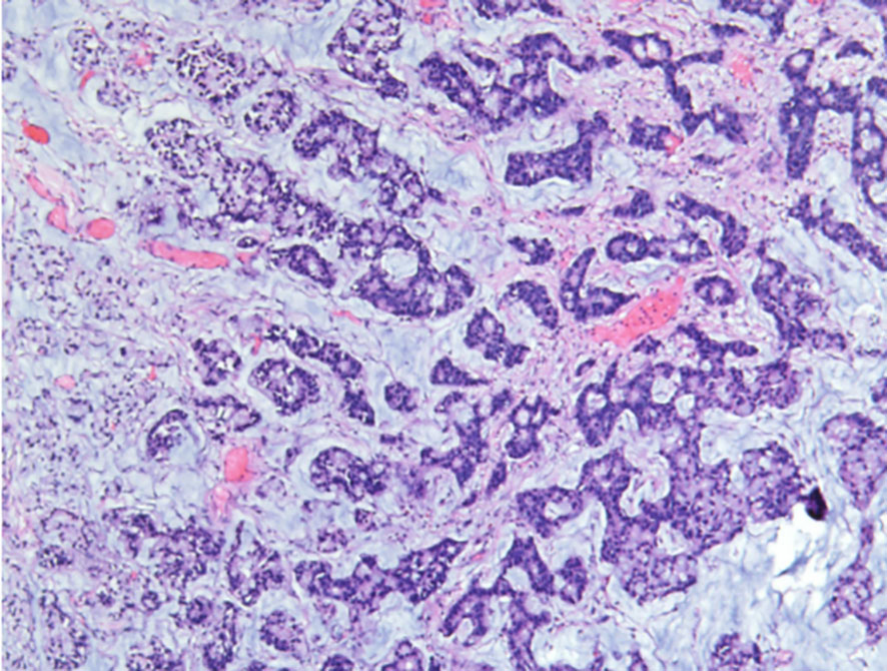
HE-stained section of the primary mucinous adenocarcinoma of the bladder.

**Figure 2:**
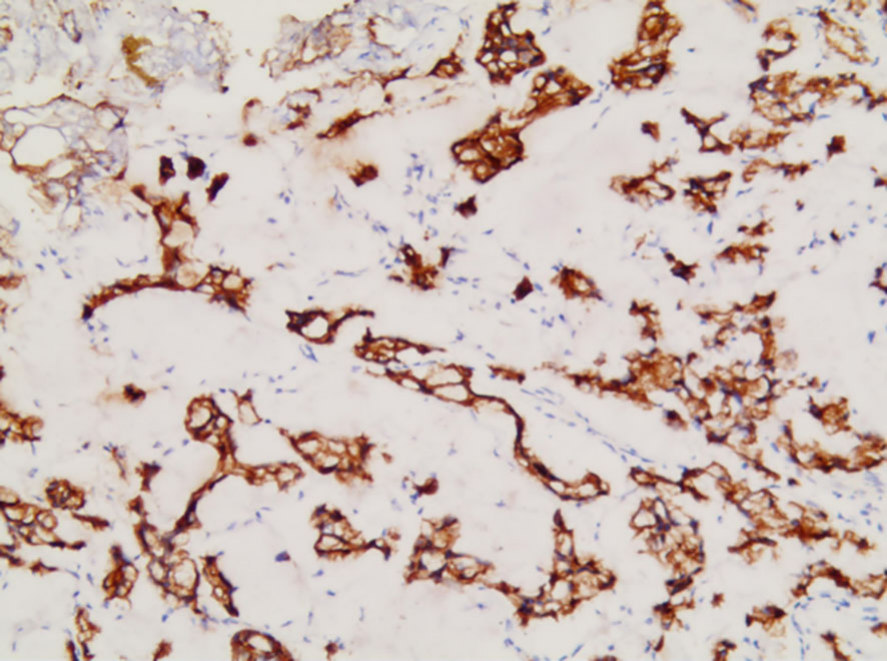
CK20 (+).

**Figure 3:**
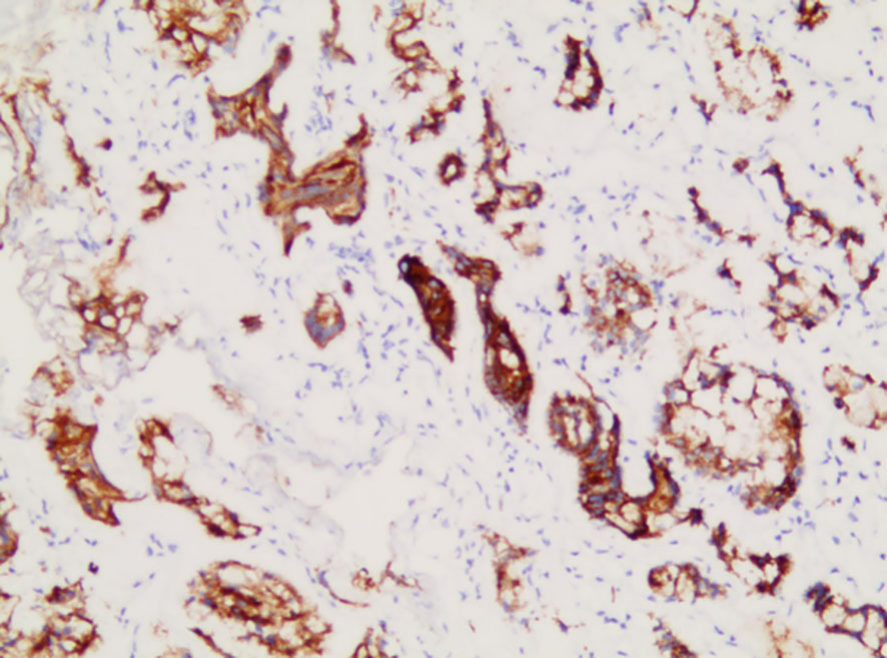
EMA (+).

A 70-year-old female patient was admitted to our hospital on June 3, 2024, with a 3-month history of progressive urinary difficulty accompanied by urinary frequency, urgency, and diminished urine stream. Gross hematuria was absent. The patient’s general condition was stable at admission. Physical examination revealed no tenderness in the bilateral renal areas, no suprapubic distension or tenderness, and no abnormalities of the external urethral meatus. Auxiliary examinations: Urinalysis showed 0 RBCs/HP and 49.9 WBCs/HP. The complete blood count and tests for liver and kidney function revealed no major abnormalities. AFP, CEA, CA-125, and CA19–9 were all within the normal ranges. The CT(computed tomography) scan of the lower abdomen with contrast demonstrated: a heterogeneously enhancing soft tissue mass at the bladder neck, with a heterogeneously enhancing soft tissue mass lesion spanning approximately 2.5 x 2.8 x 1.2 cm. The chest CT and contrast-enhanced abdominal CT scans showed no evidence of metastasis. Based on these findings, the patient underwent transurethral resection of bladder tumor on June 6, 2024. Intraoperative findings revealed a circumferential, cauliflower-like papillary tumor predominantly involving the anterior half of the bladder neck (10 o’clock to 2 o’clock positions). An additional papillary lesion was identified approximately 1 cm proximal to the urethral orifice. Immunohistochemical analysis of the resected specimen demonstrated the following: CK7 (–), CK20 (+), Ki-67 (≈60%+), EMA (+), and CDX-2 (+) (**Figures 1**–**3**). **Figure 1** shows abundant extracellular mucin pools and malignant glandular structures on HE staining. **Figure 2** demonstrates diffuse cytoplasmic CK20 positivity, and **Figure 3** shows membranous EMA expression in tumor cells. These findings were consistent with a glandular neoplasm of intestinal phenotype. The histopathological diagnosis was mucinous adenocarcinoma of the bladder. To exclude the possibility of metastatic adenocarcinoma originating from the gastrointestinal tract, the patient underwent esophagogastroduodenoscopy and colonoscopy postoperatively. Both examinations revealed no evidence of primary gastrointestinal malignancy.

One month after the initial TURBT, the patient underwent robot-assisted laparoscopic radical cystectomy with bilateral ureterostomy and pelvic lymph node dissection at another hospital. Postoperative pathological examination of the radical cystectomy specimen confirmed the diagnosis of mucinous adenocarcinoma of the bladder, with tumor invasion extending beyond the bladder wall into the uterus (pT4a according to the AJCC 8th edition staging system). None of the examined lymph nodes showed metastatic involvement (pN0). No distant metastases were identified (M0). The overall pathological stage was pT4aN0M0. Focal carcinoma *in situ* was observed at the urethral resection margin. Immunohistochemistry findings: CK7 (–),CDX-2 (+), CEA (+), CK20 (+), Villin (+), β-catenin (membrane+), SATB2 (-), GATA3 (-), P53 (+), Ki-67 (approximately 70%+). The patient did not receive adjuvant radiotherapy or chemotherapy postoperatively for personal reasons.

The patient has been followed up regularly at the urology outpatient clinic. At 6 months postoperatively, follow-up assessment included physical examination, serum tumor markers (CEA, CA19-9), and contrast-enhanced CT of the chest, abdomen, and pelvis. The CT scan showed no evidence of local recurrence or distant metastasis in the lungs, liver, or retroperitoneal lymph nodes, and no complications such as hydronephrosis or ureteral stricture were noted. The patient continues to be followed with regular imaging and laboratory surveillance.

## 3 Discussion

Distinguishing primary bladder mucinous adenocarcinoma (BMA) from metastatic adenocarcinoma, particularly of colorectal origin, remains a persistent diagnostic dilemma. This challenge stems from the substantial morphological and immunohistochemical overlap between these two entities (4). Accurate differentiation carries crucial therapeutic and prognostic consequences, as the management of a primary bladder neoplasm is fundamentally different from that of a metastatic gastrointestinal malignancy.

Bladder adenocarcinoma often manifests with nonspecific symptoms, most commonly hematuria, dysuria, and suprapubic discomfort. These vague clinical presentations, together with the inherently low incidence of the disease, frequently result in a delayed clinical diagnosis (5). Regarding tumor location, the trigone and posterior wall represent the most commonly affected sites, although lesions can arise elsewhere in the bladder, as was observed in our patient. A comprehensive staging evaluation is essential and should include contrast-enhanced computed tomography (CT) of the chest, abdomen, and pelvis to delineate the local extent of disease, assess lymph node involvement, and detect distant metastases.

A critical step in the diagnostic workup of any suspected primary bladder adenocarcinoma is the exclusion of a metastatic gastrointestinal primary. All patients should undergo upper and lower gastrointestinal endoscopy, as was performed in our case. Notably, the patient’s postoperative gastroscopy and colonoscopy revealed no evidence of a primary gastrointestinal malignancy, providing the first line of evidence against metastatic disease.

Immunohistochemistry plays a central role in the distinction between primary bladder adenocarcinoma and metastatic colorectal adenocarcinoma. Although both entities can express CK20, CDX-2, and Villin, differential staining patterns for CK7, SATB2, β-catenin, and GATA3 provide additional diagnostic clues. Roy et al. reported that CK20 was positive in all 24 primary bladder adenocarcinomas studied, whereas CK7 positivity was observed in only 33.3% of cases (4). Wang et al. similarly found CK7 positivity in approximately 65% of primary bladder adenocarcinomas and CK20 positivity in 53%. Collectively, these data indicate that CK7 and CK20 alone do not reliably distinguish primary from secondary adenocarcinomas (8).

SATB2 is a nuclear protein expressed in colorectal and appendiceal neoplasms, and its utility in distinguishing colorectal from bladder primary has been investigated. Giannico et al. examined SATB2 expression in 43 primary bladder/urinary tract adenocarcinomas and 22 colorectal adenocarcinomas, reporting positivity in 49% and 77% of cases, respectively (9). SATB2 positivity does not conclusively rule out a bladder primary, owing to the moderate rate of expression of this marker in such tumors. In contrast, a negative SATB2 result—as was observed in our patient—favors a primary bladder origin, particularly when interpreted in conjunction with additional clinical and immunohistochemical features.

The subcellular localization of β-catenin provides the greatest discriminatory power in this differential diagnosis. Wang et al. reported that nuclear β-catenin expression was detected in 13 of 16 (81%) colorectal adenocarcinomas that secondarily involved the bladder (8). By contrast, none of the primary bladder adenocarcinomas examined exhibited nuclear staining. Instead, these tumors demonstrated membranous β-catenin expression, occasionally accompanied by cytoplasmic positivity. The membranous staining pattern observed in our case is therefore consistent with a primary bladder origin.

GATA3, a key marker for urothelial differentiation, was negative in our patient. This finding effectively excludes the possibility of conventional urothelial carcinoma and its glandular variant. Integrating the endoscopic findings, negative gastrointestinal workup, and the collective immunohistochemical profile—CK7(–), CK20(+), CDX-2(+), Villin(+), β-catenin(membrane+), SATB2(–), GATA3(–)—the diagnosis of primary mucinous adenocarcinoma of the bladder was firmly established.

Currently, no standardized clinical guidelines specifically address the management of primary bladder mucinous adenocarcinoma. Given its rarity and aggressive behavior, clinical decisions are largely derived from limited case reports and small retrospective series. Radical cystectomy (RC) with bilateral pelvic lymph node dissection is generally considered the standard surgical approach for localized disease. The EAU-ESMO consensus statements on variant bladder cancer recommend RC with lymphadenectomy as the treatment of choice for non-metastatic primary bladder adenocarcinoma, acknowledging the limited evidence base (10).

For patients with locally advanced disease (pT3–pT4) or positive lymph nodes, adjuvant chemotherapy may be considered, drawing upon regimens established for colorectal adenocarcinoma due to the morphological and immunohistochemical similarities between these tumor types. Commonly referenced regimens include FOLFOX and XELOX. Wang et al. described the use of FOLFOX-6 as adjuvant therapy following radical surgery in a patient with bladder outlet mucinous adenocarcinoma (11). Kiyama et al. reported a case of pT4aN2M0 primary bladder mucinous adenocarcinoma treated with robotic-assisted radical cystectomy followed by three months of adjuvant XELOX (12). with the patient remaining progression-free at eight months postoperatively. Nonetheless, the efficacy of adjuvant chemotherapy for BMA has not been validated in prospective trials. Therefore, treatment decisions should be guided by patient-specific factors, including age, performance status, tumor stage, resection margin status, and patient preference.

In our case, although the patient had pT4a disease and a positive urethral resection margin, adjuvant chemotherapy was not administered. This decision was made after a thorough discussion between the multidisciplinary team and the patient, who was informed of the uncertain benefit of adjuvant therapy for this rare tumor subtype and the potential toxicities associated with systemic chemotherapy. The patient ultimately declined adjuvant treatment for personal reasons.

Regarding non-surgical therapeutic options, the role of systemic therapy for BMA remains investigational. A recent case report described a patient with metastatic BMA who showed significant uptake on 99mTc-FAPI-46 scintigraphy, suggesting potential suitability for FAPI-targeted radioligand therapy, though the patient succumbed to disease before treatment could be initiated (13). Additionally, approximately 20%–30% of bladder adenocarcinomas express PD-L1, raising the possibility of immune checkpoint inhibitor therapy (14). However, prospective studies are urgently needed to establish the safety and efficacy of these emerging approaches.

The prognosis of primary bladder adenocarcinoma is generally poor, reflecting its aggressive biological behavior and frequent late-stage presentation at diagnosis. Using the Surveillance, Epidemiology, and End Results (SEER) database, Natale et al. analyzed 2305 cases of primary bladder adenocarcinoma and reported overall survival rates of 54.8% at 2 years, 36.1% at 5 years, and 25.4% at 10 years. Disease-specific survival rates were 62.0% at 2 years, 47.1% at 5 years, and 40.1% at 10 years (15). The mucinous subtype, along with signet-ring cell and unclassified histologies, was associated with worse outcomes compared to papillary and other variants. It is important to note, however, that these survival estimates derive from heterogeneous patient populations and historical treatment eras, and thus may not fully reflect contemporary outcomes for patients managed with modern surgical and oncologic approaches.

**Table 1** summarizes the clinical characteristics, treatment approaches, and outcomes of all reported cases of primary bladder mucinous adenocarcinoma over the past decade, including our patient. Compared with the 10 cases listed in **Table 1** (from Li et al., 2025 (16); Wang et al., 2020 (17); Kiyama et al., 2024 (12); Maja et al., 2022 (18); Cheng et al., 2025 (6); Wang et al., 2022 (11); Ghewade et al., 2024 (19); Vijayakumar et al., 2020 (20), plus our case), several observations can be made.

**Table 1:** Case data of primary bladder mucinous adenocarcinoma patients reported in the past decade.

Literature	Age (years)	Gender	Clinical symptoms	Tumor location	Tumor size	Treatment method	Immunohistochemistry	Prognosis
Li Xiang et al. (16)	63	Male	Gross hematuria	NA	NA	Transurethral resection of bladder tumor (TURBT), laparoscopic radical cystectomy + bilateral ureterostomy	CK-P, CK7, CK20, CEA, CDX-2, β-catenin, P53 (+), PAX-8, GATA-3, PAS (–)	Follow-up at 9 months showed no distant metastasis or recurrence
Wang Qunfeng et al. (17)	54	Male	Right flank discomfort and soreness	Multiple lesions in the bladder trigone area	Multiple lesions, largest diameter approximately 1.3 cm	Transurethral resection of bladder tumor (TURBT), laparoscopic radical cystectomy with ileal conduit	NA	No tumor recurrence at 18-month follow-up
Vijayakumar et al. (12)	48	Male	Painless gross hematuria	Bladder trigone and right lateral wall	NA	Transurethral resection of bladder tumor (TURBT), robot-assisted laparoscopic radical cystectomy with ileal conduit, XELOX chemotherapy regimen	CK20, CDX-2, SATB2, β-catenin (+), CK7, NKX3 (-)	No tumor recurrence at 11-month follow-up
Maja et al. (18)	75	Female	Gross hematuria	Right bladder wall	2×2 cm	Transurethral resection of bladder tumor (TURBT), laparoscopic partial cystectomy	CK20, CDX-2, CK7, β-catenin (+)	Follow-up for 2 years showed no tumor recurrence or distant metastasis
Cheng et al. (6)	72	Male	Painless gross hematuria	Posterior left wall of the bladder	NA	Transurethral resection of bladder tumor (TURBT), robot-assisted laparoscopic radical cystectomy with ileal conduit, FOLFOX chemotherapy regimen	CK20, CK7, GATA3, SATB2, Villin (-) CDX-2, β-catenin, Ki67 (≈60%)	No tumor recurrence at 6-month follow-up
Wang et al. (11)	62	Female	Urinary retention	Near the urethra of the bladder	5.6 × 4.4 cm	Laparoscopic radical cystectomy + bilateral ureterostomy, FOLFOX chemotherapy regimen	CKpan, CK20, Ki-67, Syn, CD56, CgA (+), GATA-3, CDX2 (-)	No tumor recurrence at 60-day follow-up
Ghewade et al. (19)	44	Female	Gross hematuria	Bladder base	10.8 × 9 × 8 cm	Transurethral resection of bladder tumor (TURBT), laparoscopic radical cystectomy with ileal conduit	CK20, CK7 (+)	Lost to follow-up
Vijayakumar et al. (20)	63	Male	Suprapubic pain	Bladder base and bladder neck	NA	Transurethral resection of bladder tumor (TURBT), laparoscopic radical cystectomy with ileal conduit	CK20 (+), CDX-2, GATA-3 (-)	Lost to follow-up

Regarding age and gender, the patients ranged from 44 to 75 years (median 62.5 years), with a male-to-female ratio of 6:5, showing no strong sex predilection. Gross hematuria remained the most common presenting symptom, reported in 70% (7/10) of cases, whereas our patient presented with voiding symptoms without hematuria—a less typical presentation. In terms of treatment, radical cystectomy with pelvic lymph node dissection was performed in 9 of the 11 cases (82%), while TURBT alone was performed in only one case, where it was followed by radical cystectomy after recurrence. Among the 11 cases, four patients received adjuvant chemotherapy (36%), predominantly with FOLFOX or XELOX regimens, and two patients received adjuvant radiotherapy. Our patient did not receive adjuvant therapy, primarily due to her personal decision after being informed of the uncertain benefit for this rare tumor.

Regarding oncologic outcomes, among the 11 cases, 8 (73%) reported no evidence of recurrence or metastasis during follow-up periods ranging from 6 to 24 months. Three cases were lost to follow-up or had incomplete outcome reporting. Our patient has remained disease-free at 6 months of follow-up, consistent with the short-term outcomes described in most recent reports. However, longer follow-up is needed given the aggressive nature of this tumor and its propensity for late recurrence.

These comparisons illustrate the generally favorable short-term outcomes achieved with radical cystectomy for localized BMA. At the same time, they reveal substantial variability in decisions regarding adjuvant therapy—an inconsistency that stems from the lack of standardized guidelines—and emphasize the critical need for systematic long-term follow-up data.

## 4 Conclusion

In summary, primary mucinous adenocarcinoma of the bladder is a rare but highly aggressive malignancy that poses significant diagnostic and therapeutic challenges. Accurate diagnosis requires the integration of endoscopic evaluation with a carefully selected panel of immunohistochemical markers—particularly including SATB2 and β-catenin—to confidently exclude metastatic adenocarcinoma, especially from colorectal origin. Treatment decisions should be individualized based on tumor stage, resection margin status, patient age and performance status, and patient preference, with radical cystectomy and pelvic lymph node dissection representing the cornerstone of curative-intent therapy for localized disease. The absence of standardized treatment guidelines, the lack of high-level evidence for adjuvant therapy, and the emergence of potential novel diagnostic approaches such as FAPI-targeted imaging underscore the urgent need for multicenter prospective studies and international collaborative efforts to establish evidence-based diagnostic and therapeutic protocols for this rare cancer.

## Data Availability

The original contributions presented in the study are included in the article/supplementary material. Further inquiries can be directed to the corresponding author.

## References

[1] Lu H Zhu W Mao W Zu F Wang Y Li W . Trends of incidence and prognosis of primary adenocarcinoma of the bladder. Ther Adv Urol. (2021) 13:17562872211018006. doi: 10.1177/17562872211018006. PMID: 34104222 PMC8150450

[2] Kadouri Y Hachem F Lakssir J Sayegh H Benslimane L Nouini Y . Primitive adenocarcinoma of the bladder: About 6 cases. Pan Afr Med J. (2020) 36:61. doi: 10.11604/pamj.2020.36.61.20176. PMID: 32733631 PMC7371441

[3] Yamamoto S Ashida S Nao T Murakami K Iga R Shimamoto T . Comprehensive genomic profiling of primary bladder mucinous adenocarcinoma, a rare genitourinary cancer: A case report. Urol Case Rep. (2025) 58:102892. doi: 10.1016/j.eucr.2024.102892. PMID: 39660097 PMC11629251

[4] Roy S Smith MA Cieply KM Acquafondata MB Parwani AV . Primary bladder adenocarcinoma versus metastatic colorectal adenocarcinoma: a persisting diagnostic challenge. Diagn Pathol. (2012) 7:151. doi: 10.1186/1746-1596-7-151. PMID: 23121893 PMC3502416

[5] Santa F Akgul M Tannous E Pacheco RR Lightle AR Mohanty SK . Primary adenocarcinoma of the urinary tract and its precursors: Diagnostic criteria and classification. Hum Pathol. (2025) 155:105734. doi: 10.1016/j.humpath.2025.105734. PMID: 39988060

[6] Cheng X Wang W Yang L Tai S Tao J Fu Y . Diagnosis and therapeutic strategies for primary bladder mucinous adenocarcinoma: a case report and literature review. Front Oncol. (2025) 15:1652375. doi: 10.3389/fonc.2025.1652375. PMID: 41132737 PMC12540155

[7] Abdallah C Mehdi M Houcem H Nizar C Samir G Mohamed D . Bladder adenocarcinoma with intestinal-type glands: A case report. Urol Case Rep. (2024) 55:102765. doi: 10.1016/j.eucr.2024.102765. PMID: 38957664 PMC11217605

[8] Wang H Lu D Yerian L Alsikafi N Steinberg G Hart J . Immunohistochemical distinction between primary adenocarcinoma of the bladder and secondary colorectal adenocarcinoma. Am J Surg Pathol. (2001) 25:1380–7. doi: 10.1097/00000478-200111000-00005. PMID: 11684954

[9] Giannico GA Gown AM Epstein JI Revetta F Bishop JA . Role of SATB2 in distinguishing the site of origin in glandular lesions of the bladder/urinary tract. Hum Pathol. (2017) 67:152–9. doi: 10.1016/j.humpath.2017.07.002. PMID: 28711650

[10] Witjes JA Babjuk M Bellmunt J Bruins HM De Reijke TM De Santis M . EAU-ESMO consensus statements on the management of advanced and variant bladder cancer—an international collaborative multistakeholder effort†: Under the auspices of the EAU-ESMO guidelines committees. Eur Urol. (2020) 77:223–50. doi: 10.1016/j.eururo.2019.09.035. PMID: 31753752

[11] Wang D Zhang K Guan L Wen N . Imaging features of primary mucinous adenocarcinoma of bladder outlet and urethra: a case report and literature review. Trans Cancer Res. (2022) 11:2416–24. doi: 10.21037/tcr-22-1547. PMID: 35966298 PMC9372207

[12] Kiyama Y Sekii Y Inoguchi S Matsumura S Kitakaze H Hongo S . A case of primary adenocarcinoma mucinous subtype of the bladder. Hinyokika Kiyo Acta Urologica Japonica. (2024) 70:89–92. doi: 10.14989/ActaUrolJap_70_4_89 38965907

[13] Raeisi N Saber Tanha A Khooei A Ataei N Aghaee A . Favorable 99mTc-FAPI-46 uptake in primary mucinous adenocarcinoma of the bladder. Clin Nucl Med. (2025) 51:e55–6. doi: 10.1097/RLU.0000000000005933 40357621

[14] Jones D Guan JJ Calagua C Hansel DE Epstein JI Ye H . Primary adenocarcinoma of the bladder lacks mismatch repair deficiency and demonstrates PD-L1 expression in tumor-infiltrating immune cells, with implications in both diagnosis and therapeutics. Hum Pathol. (2019) 94:58–63. doi: 10.1016/j.humpath.2019.10.005. PMID: 31666198

[15] Natale C Leinwand GZ Chiang J Silberstein JL Krane LS . Reviewing the demographic, prognostic, and treatment factors of primary adenocarcinoma of the bladder: a SEER population-based study. Clin Genitourin Cancer. (2019) 17:380–8. doi: 10.1016/j.clgc.2019.06.010. PMID: 31395362

[16] Li X Yang XB Liang HW Liu Q . A case report of primary bladder mucinous adenocarcinoma combined with signet-ring cell carcinoma. J Contemp Urol Reprod Oncol. (2025) 17:58–9. doi: 10.3870/j.issn.1674-4624.2025.01.015

[17] Wang QF Liang CZ Zhu JS Chen ZJ Dai YH Bao T . A case of bladder mucinous adenocarcinoma. J Contemp Urol Reprod Oncol. (2020) 12:116–28. doi: 10.1515/biol-2019-0064. PMID: 33817194 PMC7874758

[18] Maja SG Slavica KK Suad A Rubens J . Bladder mucinous adenocarcinoma as a diagnostic challenge: a case report. Pan Afr Med J. (2022) 42:221. doi: 10.11604/pamj.2022.42.221.35032. PMID: 36845250 PMC9949276

[19] Ghewade P Shukla S Vagha S Kalode SS Gadkari P . Primary adenocarcinoma of the urinary bladder: a case report on a rare Malignancy. Cureus. (2024) 16:e66269. doi: 10.7759/cureus.66269. PMID: 39238745 PMC11375912

[20] Vijayakumar V Natesan G Sudhakar M Sudhakar M Prakasam U Seeralan V Kaliyaperumal M . Primary mucinous adenocarcinoma of the bladder: Case report and review of literature. Indian J Surg Oncol. (2020) 11:44–7. doi: 10.1007/s13193-019-01028-y PMC7534744 33088128

